# The effect of bovine leukemia virus on dairy cow longevity

**DOI:** 10.3168/jdsc.2021-0187

**Published:** 2022-03-03

**Authors:** Oscar J. Benitez, Rebecca M. LaDronka, Bo Norby, Daniel L. Grooms, Paul C. Bartlett

**Affiliations:** 1 Department of Large Animal Clinical Sciences, College of Veterinary Medicine, Michigan State University, 736 Wilson Rd, East Lansing 48895; 2 College of Veterinary Medicine, Iowa State University, 2420 Lincoln Way, Ames 50014

## Abstract

Bovine leukemia virus (BLV) is a retrovirus of cattle that infects approximately 45% of all US dairy cattle, with about 90% of US dairy herds having at least one infected animal. Studies have found BLV infection to be associated with multiple measures of decreased immune function, which may explain the observed economic losses from milk production, decreased cow longevity, and predisposition to lymphoma and other diseases. Our objective was to measure the association between BLV infection and cow longevity in dairy cow operations. Ninety-one dairy herds from 9 US states volunteered to participate in this study. Milking dairy cows (n = 3,611) were tested for BLV antibodies using an ELISA milk test, and their presence in the herd was monitored for an average of 29 mo. The survival analysis controlled for herd and lactation number. Cows sold for dairy purposes were excluded, and individual cow results were not shared with producers so as not to influence culling decisions. Overall, 47.1% (1,701/3,611) of cows were BLV-positive by ELISA. The significant hazard ratio of 1.30 indicated that positive cows were 30% more likely than their negative herdmates to die or be culled during the monitoring period. These results are consistent with other studies in finding a negative effect of BLV infection on cow lifespan.

## Highlights

• Dairy cows were tested for BLV with an ELISA milk test and followed for survival.
• BLV-positive cows had shortened lifespans compared with BLV-negative herdmates.
• BLV ELISA results were not associated with producer-reported reasons for culling.

## Graphical Abstract

**Figure:**
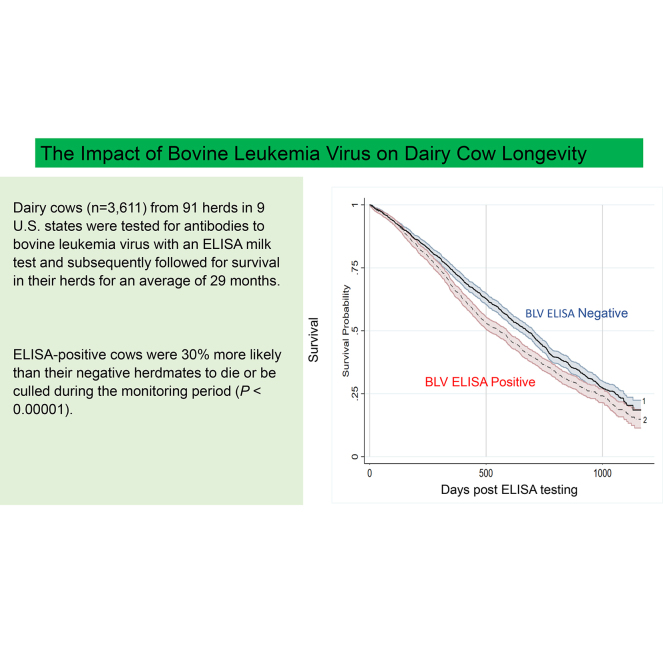
**Summary:** Dairy cows (n = 3,611) from 91 herds in 9 US states were tested for antibodies to bovine leukemia virus (BLV) with an ELISA milk test and subsequently followed for survival in their herds for an average of 29 mo. Controlling for age and herd effects, ELISA-positive cows were 30% more likely (*P* < 0.00001) than their negative herdmates to die or be culled during the monitoring period. Measures of the total economic impact of this virus on cattle production must include an assessment of any shortened lifespan.

In the 1970s, about 10% of US dairy cattle were seropositive for bovine leukemia virus (**BLV**) compared with about 45% today (Bartlett et al., 2014; LaDronka et al., 2018). Most BLV-infected animals remain asymptomatic and act as carriers of the virus, and about 30% of infected cattle develop a persistent lymphocytosis. Less than 5% progress to the fatal, clinical form of BLV infection: lymphoma (Tajima et al., 1998; USDA, 2007; Bartlett et al., 2013, 2020). Infection with BLV alters the cow's immune function by disrupting immune cell signaling molecules, cytokine production, self-destruction of activated infected cells, and irregularity of immune cell proliferation and apoptosis and typical lymphocyte ratios (Della Libera et al., 2015; Frie and Coussens, 2015; Swenson et al., 2013). Likely as a result, BLV-infected cattle appear to be more susceptible to a variety of diseases that negatively affect their production performance and longevity or lifespan (Frie and Coussens, 2015; Blagitz et al., 2017).

The major economic impacts of BLV are decreased milk production, shortened longevity, and lymphoma (Bartlett et al., 2014, 2020). A sophisticated economic analysis is needed to estimate the total economic impact because these 3 cost components undoubtedly overlap and interact. In this report, we will focus on the impact of BLV infection on cow longevity (lifespan).

Epidemiologic studies in dairy cattle have demonstrated an association between BLV infection and decreased productive lifespan within herds (Thurmond et al., 1985; Bartlett et al., 2013; Nekouei et al., 2016), although others have not found this association (Huber et al., 1981; Rhodes et al., 2003; Tiwari et al., 2005). More studies from diverse dairy cow populations are needed to provide additional and better estimates of the economic impact of BLV to determine whether controlling this disease is cost effective. In 2013, our research group published the results of a BLV survival analysis in Michigan (Bartlett et al., 2013), which showed a 23% increase in culling when BLV ELISA-positive cows were compared with herdmates of the same age. However, this 2013 study was conducted among selected dairy herds in only one state (Michigan) and confirmation is necessary. The main objective of the current study was to measure the effect of BLV on cow longevity using a more extensive database, including herds of all sizes from across different regions of the United States.

The use of animals in this study was approved by the Michigan State University (East Lansing) Animal Care and Use Committee. For the current study, dairy herds were enrolled by DHI technicians who volunteered with their client herds to participate in this study (LaDronka et al., 2018). We studied 91 herds (3,611 cows) in 9 states: 33 herds in the East (New York, Pennsylvania, Vermont, and North Carolina), 56 herds in the Midwest (Minnesota, Wisconsin, Michigan, and Ohio), and 2 herds in the South (Texas). A total of 3,611 individual cow milk samples were tested for milk BLV ELISA antibodies. Holstein cows made up 94% of the tested animals; the remainder were crossbred (4%), Jersey (2%), and other breeds including Brown Swiss and Guernsey (<1%).

With the exception of cows calving <10 d previously, DHI-collected milk samples from the 10 most recently calved cows were tested by BLV ELISA from cows in the first, second, third, and fourth and greater lactations in each herd. Small herds with <10 cows per lactation necessarily had fewer than 40 milk samples tested. As such, neither the producer nor the technician selected which animals would be sampled. Thereafter, DHI records were followed forward in time to assess survival in the herd. Individual cow results were not shared with producers or technicians so as to not influence culling decisions. Herds were monitored for an average 872 d (range 825 to 1,128). All cows in the same herd were monitored for the same number of days, which was determined largely by the scheduling of their milk testing visits.

Milk samples were collected via routine DHI milk sampling protocols. Samples were collected in individual vials with preservative (bronopol/natamycin) and shipped from their respective local DHI to the CentralStar Cooperative (Lansing, MI) as described in LaDronka et al. (2018). Samples were stored at room temperature, and ELISA testing was completed within 18 d from the date of sample collection. Detection of BLV antibodies in milk by ELISA has been described previously (LaDronka et al., 2018). Optical density (**OD**) values >0.1 were considered positive for previous BLV exposure. Using a cutoff of 0.1, this assay has close agreement (κ = 0.86) with serum ELISA, which has sensitivity and specificity of 99.8% and 100%, respectively, using agar gel immunodiffusion (AGID) as the reference test (Simard et al., 2000; Walsh et al., 2013).

Survival analysis was conducted using Stata software (version 15.1; StataCorp LP) using nonparametric Kaplan-Meier survival graphs and semi-parametric Cox proportional hazard models handling ties with the Breslow method. Kaplan-Meier graphs were visual representations that assisted in interpretation of the data. Shared frailty (random effect) of the herd was included. Results were considered statistically significant at *P* < 0.05. Days at risk for culling were calculated by subtracting the date of milk sampling from the date of culling or censoring. The main effects were estimated after controlling for lactation number (1, 2, 3, and >4). Additionally, the first-order interactions between BLV OD status and lactation were examined. The shared frailty model using multiplicative gamma-distributed random effects on the hazard scale was used to model within-herd correlation among cows. Cox-Snell and Schoenfeld residuals and the interaction between the log-transformed time-to-event and all predictor variables were used to test model fit (Cleves et al., 2010).

A total of 1,701 (47.1%; 95% CI: 45.4–48.7%) cows tested positive for BLV antibodies, and 1,910 tested negative. During the monitoring period, 63.5% (95% CI: 61.8–65.1) of the cows were culled or died and 36.5% (95% CI: 34.8–38.1) remained in the herd (Table 1). Of the cows that tested positive by milk ELISA, 1,124 (51.8%; 95% CI: 49.7–53.9%) left the herd during the study period compared with 1,044 cows (48.2%; 95% CI: 46.1–50.3%) that tested negative for BLV antibodies in milk. The survival analysis showed a statistically significant difference in the survival of the cows based on milk-ELISA status (*P* < 0.00001; Table 1, Figure 1). Cows positive by ELISA faced a 30% greater hazard of being culled than their BLV-negative herdmates. The hazard ratios increased progressively with lactation (Table 1), with cows in fourth or greater lactation having an almost 3-fold-greater risk of leaving the herd. However, the interaction between BLV infection and lactation was not statistically significant (*P* > 0.05), indicating that the effect of BLV on cow longevity appeared to be independent of cow age.

**Table 1:** Survival of cattle following bovine leukemia virus (BLV) testing (negative or positive) for milk BLV antibodies by ELISA^1^

Predictor	Hazard ratio	*P*-value	95% CI
Negative (OD^2^ <0.1)	—	—	—
Positive (OD >0.1)	1.30	<0.001	1.19–1.43
Lactation 1	—	—	—
Lactation 2	1.51	<0.001	1.33–1.73
Lactation 3	1.95	<0.001	1.71–2.23
Lactation 4+	2.90	<0.001	2.55–3.31

1 Average length of follow-up was 29 mo.

2 Optical density.

**Figure 1:**
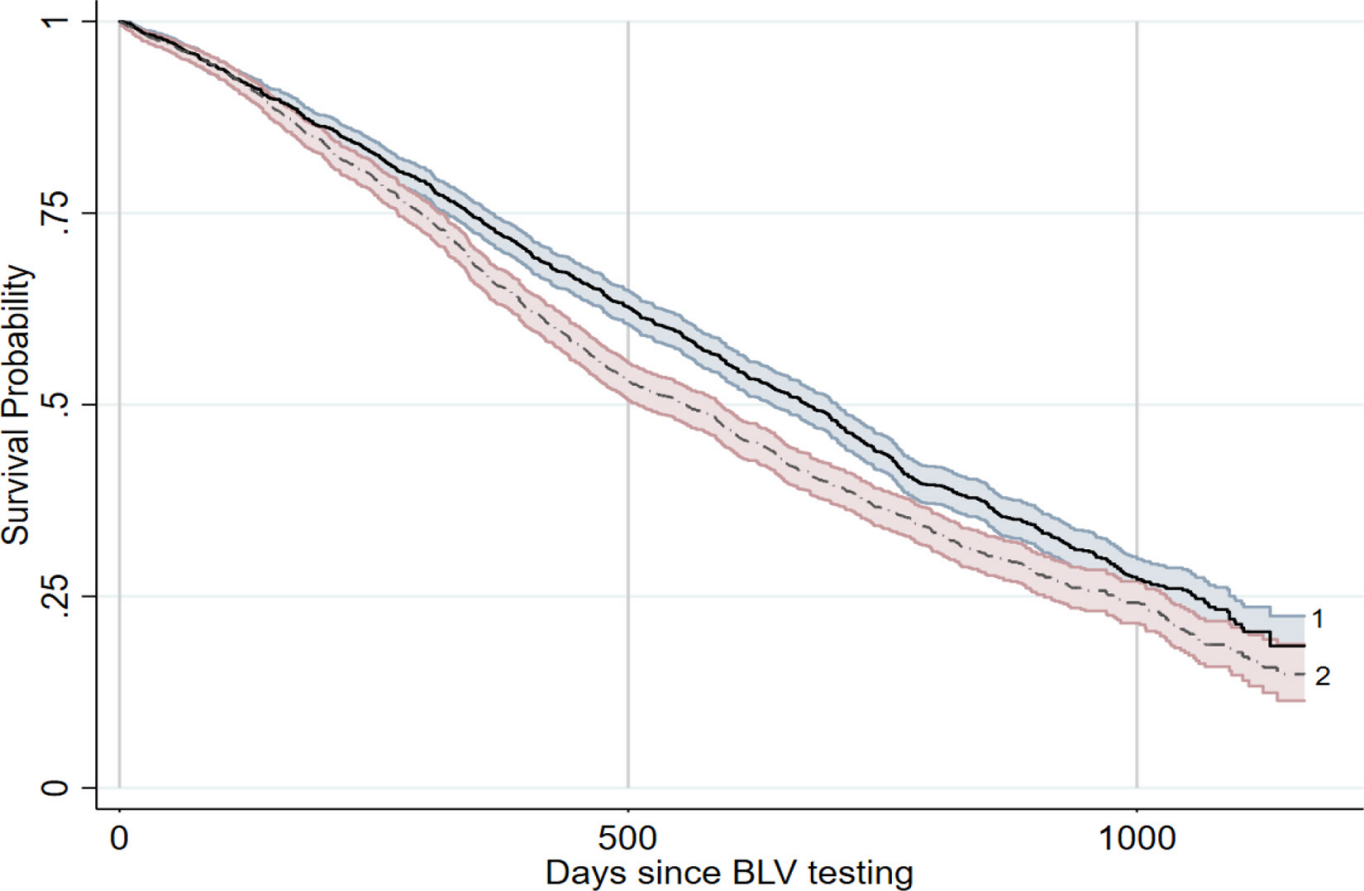
Cumulative survival of cattle following bovine leukemia virus (BLV) ELISA testing, showing the proportion of cattle surviving (y-axis) and days since BLV testing (x-axis). Curve 1 = BLV-negative cows and curve 2 = BLV-positive cows; shaded bands indicate 95% CI. Survival data were collected for an average of 29 mo after ELISA testing. The significant hazard ratio of 1.30 indicates that BLV-positive cows were 30% more likely than their BLV-negative herdmates to die or be culled during the monitoring period.

Nonsignificant *P*-values (*P* > 0.05) for time-varying interactions between log-time, BLV status (or OD categories), and lactation number indicated that the basic assumptions of proportional hazards were not violated. Visual assessment of the cumulative hazard function H(t) plotted against the Cox-Snell residuals indicated a good fit of the model.

The DHI categories for animals leaving the herd showed that the “found dead” category was indicated at a higher percentage for ELISA-positive cows than for ELISA-negative cows (risk ratio = 1.15; 95% CI: 1.02–1.29), but no other categories were significantly associated with the presence of BLV antibodies.

In a study of beef cows, it was recently reported that BLV-infected beef cows with a high BLV proviral load in blood were 84% more likely to be culled or die compared with their BLV-negative herdmates (Benitez et al., 2020). Together, these studies (Bartlett et al., 2013; Benitez et al., 2020; current study) support the association of BLV infection with reduced bovine lifespan. Other epidemiological studies in the United States (Thurmond et al., 1985) and Europe (Emanuelson et al., 1992) also suggest a negative relationship between BLV infection and cow longevity.

By including herd and lactation number in our statistical analysis, we enabled comparison of BLV antibody-positive dairy cows with their negative herdmates of the same age and controlled for potential confounding effects of herd-level management factors such as herd size and type of housing.

We likely underestimated the impact of BLV infection on cow survival because some cows that tested negative would have become infected later during the monitoring period. This misclassification bias would have diminished the observed difference in cow longevity associated with ELISA results because the BLV-negative group included some cows that were infected, albeit relatively recently, after their BLV ELISA test. Very few false-negative tests should have occurred because antibody production usually occurs within a few weeks of infection.

In conclusion, the presence of BLV antibodies in milk was associated with a 30% reduction in cow longevity (lifespan) over an average 29-mo follow-up period. Future survival analysis field studies should confirm and more fully describe the association between BLV infection and cow longevity in different cattle breeds, geographic areas, and management systems, perhaps supported by additional BLV diagnostics such as proviral load and lymphocyte count.
